# [Retracted] Tumor-released lncRNA H19 promotes gefitinib resistance via packaging into exosomes in non-small cell lung cancer

**DOI:** 10.3892/or.2025.8973

**Published:** 2025-08-12

**Authors:** Yi Lei, Wang Guo, Bowang Chen, Lu Chen, Jiaxin Gong, Weimin Li

Oncol Rep 40: 3438–3446, 2018; DOI: 10.3892/or.2018.6762

Following the publication of the above paper, it was drawn to the Editor's attention by a concerned reader that certain of the TEM microscopic data featured in Fig. 3C on p. 3443, the RIP assay data in Fig. 4A and the western blot data in Fig. 4D on p. 3444 were strikingly similar to data that had appeared in other articles written by different authors at different research institutes, which had either already been published before this article was received at *Oncology Reports*, or were submitted for publication at around the same time (one of which has subsequently been retracted).

Owing to the fact that certain of the abovementioned data had already been published prior to the receipt of this article at *Oncology Reports*, the Editor has decided that this paper should be retracted from the Journal. After contacting the authors, they accepted the decision to retract the paper. The Editor apologizes to the readership for any inconvenience caused.

